# FIGO–IPPS consensus statement: Addressing the global unmet needs of women with chronic pelvic pain

**DOI:** 10.1002/ijgo.70093

**Published:** 2025-04-02

**Authors:** Juan Diego Villegas‐Echeverri, Magali Robert, Jorge F. Carrillo, Isabel Green, Ivo Meinhold‐Heerlein, Rukset Attar, Rachel Pope, Georgine Lamvu

**Affiliations:** ^1^ ALGIA Unidad de Laparoscopia Ginecológica Avanzada y Dolor Pélvico Pereira Colombia; ^2^ Cumming School of Medicine Calgary Canada; ^3^ Orlando Healthcare System Orlando Florida USA; ^4^ Mayo Clinic Rochester Minnesota USA; ^5^ Justus Liebig University and University Hospital Giessen Giessen Germany; ^6^ Yeditepe University Istanbul Turkiye; ^7^ University Hospitals Cleveland Medical Center Cleveland Ohio USA; ^8^ University of Central Florida Orlando Florida USA

**Keywords:** chronic pelvic pain, gender inequality, reproductive health disparities, underserved populations, women's health policy

## Abstract

Chronic pelvic pain (CPP), a debilitating condition affecting an estimated 25% of women worldwide, represents a significant yet understudied global health crisis. Existing research is limited and likely fails to capture the true impact of CPP on global health. What is known is that CPP results in a profound health, societal, and economic burden on women. Our inability to fully understand this burden is a critical gap in women's healthcare. To address this urgent need, the International Federation of Gynecology and Obstetrics (FIGO) and the International Pelvic Pain Society (IPPS) have partnered to develop this consensus statement where we examine the key challenges to accessing CPP care and propose a framework for overcoming these barriers. We emphasize that effective global strategies for addressing the negative health consequences of living with CPP must prioritize the lived experiences of patients, empower healthcare professionals with the necessary tools and training, and drive meaningful policy change. This call to action is grounded in a comprehensive vision of women's health and rights to address the full spectrum of conditions women experience throughout their lives. Given its high prevalence, CPP must be a central focus of this expanded vision.

## 1 THE MULTIFACED PROBLEM OF CHRONIC PELVIC PAIN BEGINS WITH THE DEFINITION

Despite widespread recognition of chronic pelvic pain (CPP) as a significant clinical problem, inconsistencies in its definition across leading organizations—including the American College of Obstetricians and Gynecologists (ACOG), the International Association for the Study of Pain (IASP), WHO, and the Royal College of Obstetricians and Gynecologists (RCOG)—create substantial challenges for clinical practice, research, and education.^1^, ^2^, ^3^, ^4^, ^5^, ^6^ ACOG defines CPP based on anatomical origin (pelvic organs/structures) and duration (greater than 6 months), highlighting associated negative consequences and related symptoms.^2^ Conversely, IASP prioritizes the patient's pain experience, defining CPP with a shorter duration (3 months) and emphasizing specific pain syndromes.^7^, ^8^ WHO's ICD‐11 classification distinguishes between chronic primary pain (the condition itself) and chronic secondary pain (a symptom of an underlying condition).^8^, ^9^ The RCOG definition further complicates matters by characterizing CPP primarily as a symptom.^10^

These disparate approaches—with ACOG's broad definition contrasting with IASP and WHO's subtype categorizations, and the RCOG's focus on CPP as a symptom—hinder comparisons of research findings, the development of standardized treatment protocols, and accurate assessments of CPP's prevalence and burden.^1^, ^3^ This inconsistent overlap across definitions also complicates the training of healthcare professionals.^11^ Moreover, the frequent co‐occurrence of CPP with other conditions complicates diagnosis and often delays appropriate treatment.^12^ This heterogeneity is also reflected in the breadth and variation in the definitions for CPP. This diagnostic ambiguity creates significant challenges for researchers seeking to establish relevant outcome measures, determine accurate prevalence rates, and develop effective treatment strategies.^1^ Ultimately, the multifactorial nature of CPP, exacerbated by these definitional inconsistencies, negatively impacts the well‐being of affected individuals and impedes progress in understanding and managing this complex condition.^1^, ^3^, ^9^

## 2 THE INVISIBLE EPIDEMIC

Chronic pelvic pain affects an estimated one in four women worldwide.^1^, ^3^ Despite this high prevalence, the global burden of CPP remains understudied.^4^, ^5^ A 2006 WHO systematic review found that only 14% of low‐ and middle‐income countries (LMICs) and 51% of high‐income countries (HICs) had studies examining the prevalence of CPP.^4^ Alarmingly, this data gap persisted largely unchanged in 2014. Although gynecological diseases in women aged 25–49 years ranked seventh in the 2019 global burden of disease,^13^ this likely continues to underestimate the true impact of CPP, and we question whether this figure would be much higher if all women with CPP were included.^4^, ^6^, ^14^, ^15^

CPP remains largely invisible in global health discussions, under‐recognized, under‐researched, and under‐funded compared to other conditions with a similar prevalence, such as asthma, migraines, or chronic low back pain.^14^ This disparity highlights the urgent need to prioritize CPP.^1^ The burden substantially impacts well‐being, quality of life, productivity, and healthcare systems.^1^, ^3^, ^16^ Although disproportionately affecting women in LMICs,^4^, ^17^, ^18^ exacerbating health disparities, CPP is also under‐addressed in HICs.^4^, ^5^, ^19^, ^20^ This “invisible epidemic” is perpetuated by the following:

- Societal stigma: shame surrounding reproductive health and normalization of pelvic pain, which prevents help‐seeking.
- Healthcare provider gaps: inadequate training leads to delayed diagnosis and inadequate treatment.
- Limited research: insufficient funding hinders understanding, diagnosis, and treatment development.^11^, ^15^, ^21^

The fact that CPP primarily affects women contributes to its neglect. This gendered disparity is amplified in LMICs,^21^, ^22^, ^23^, ^24^ but even in HICs, women face discrimination, delayed diagnosis, and limited access to specialized care.^4^ Studies show that only 40% of women with CPP seek medical help,^14^, ^25^ and among those who do, many are never referred to a specialist, and nearly 50% do not receive pain therapy.^26^ Gendered differences in pain care are well‐documented, with women less likely to receive pain medication.^19^, ^21^, ^23^ These disparities are compounded for racial and ethnic minorities,^21^, ^22^ creating a cycle of underdiagnosis, inadequate treatment, and suffering, further magnified by socioeconomic factors.^19^, ^27^

## 3 THE HEALTH BURDEN AND ECONOMIC IMPACT

CPP poses a significant burden on women's lives, impacting their physical, emotional, and social well‐being.^1^, ^26^, ^28^ The persistent pain can disrupt daily activities, impairing work productivity, household responsibilities, and even basic self‐care.^24^, ^28^ Emotionally, CPP can lead to anxiety, depression, feelings of isolation, straining relationships, and diminishing quality of life.^16^, ^28^, ^29^ Many women experience pain during sexual activity, further impacting intimacy and self‐esteem.^26^, ^28^

Beyond individual suffering, CPP also strains healthcare systems and contributes to economic losses due to decreased productivity and increased medical expenses. Although the financial impact of CPP varies considerably across countries due to differences in healthcare systems, economic contexts, and research availability, the burden on society as a whole is substantial.^29^ In HICs like the USA, the estimated annual cost reaches $2.8 billion,^20^, ^29^, ^30^ while in Japan, individual women face yearly expenses in the range of ¥191 680–¥246 488.^20^, ^30^ Australian women experience annual costs in the range of $16 970–$20 898.^20^ Importantly, these figures primarily capture direct medical expenses and may not fully reflect the indirect healthcare costs not accessed by a publicly funded system.^30^, ^31^ More importantly, it does not measure the societal costs, such as lost productivity, disability, and diminished quality of life, which further amplify the economic burden.^19^, ^31^

The economic impact of CPP may also be significantly underestimated due to the consistent omission of unpaid work, typically performed by women, despite its crucial role in societal functioning.^31^ Furthermore, limited data from LMICs likely mask an even greater economic hardship faced by women in these regions.^4^, ^30^ To accurately assess the full health economic impact of CPP, including direct, indirect, and societal costs, increased research investment is essential in both HICs and LMICs.^4^, ^13^, ^19^, ^30^ A crucial component of this research must be the development of standardized methodologies for capturing indirect costs, particularly the value of unpaid work, enabling more accurate cross‐national comparisons and ultimately informing resource allocation for effective pain management.^4^, ^30^, ^31^

## 4 CHALLENGES IN ESTABLISHING CPP‐FOCUSED HEALTH POLICIES

Despite the significant global health, economic, and social impact, CPP suffers from a critical lack of recognition within health policy agendas and research funding priorities.^1^ This underinvestment hinders progress,^21^, ^23^, ^29^ perpetuating a cycle of under‐recognition, underfunding, and limited access,^31^, ^32^ resulting in unnecessary suffering and substantial economic burdens.^1^, ^3^, ^13^ The consequences are far‐reaching, resulting in unnecessary suffering for millions of women, substantial monetary burdens on individuals, families, and healthcare systems, and a significant impediment to women's overall well‐being and societal participation.^1^, ^3^, ^19^ This neglect not only reflects a general dismissal of women's health concerns but also highlights societies and policymakers' lack of awareness of the far‐reaching impacts of overlooking CPP.^1^, ^19^

To ensure that women with CPP receive the necessary care, the cycle of neglect must be broken by explicitly incorporating CPP into both local and global health agendas.^4^, ^5^, ^6^ This necessitates a commitment to increase and sustain investment in research and improve resource allocation.^1^, ^4^, ^28^ Data collection and integration into national strategies are essential to this endeavor.^1^, ^4^, ^29^ Analysis of the prevalence, burden, and economic impact of CPP will inform policy decisions and resource allocation. Therefore, CPP‐specific metrics must be developed for tracking progress in research funding and policy implementation.^1^, ^5^, ^28^, ^30^

## 5 BARRIERS TO HEALTH‐RELATED CARE

The global health community needs to prioritize a move away from the current fragmented and often inadequate approach to CPP care. Transdisciplinary care using the biopsychosocial model, while recognized as the standard of care, remains inaccessible to most women.^33^, ^34^ Admittedly, applying a biopsychosocial model to care for CPP patients can be complex and many of them experience delayed or inaccurate care due to limited access to specialists who have the proper knowledge to spearhead management strategies.^1^, ^2^, ^3^, ^33^ For instance, women with endometriosis, a common subset of the CPP population, have reported delays of 4–11 years from the onset of symptoms to diagnosis.^15^, ^35^, ^36^, ^37^ Disparities in access to quality care have been reported across LMICs and HICs, particularly for underserved populations.^4^, ^22^, ^24^, ^30^ The resulting lack of recognition and access leaves women powerless and stigmatized, leading to the overuse of surgical interventions with irreversible consequences, such as menopause and infertility.

## 6 GAPS IN HEALTHCARE PROFESSIONAL EDUCATION

Many providers lack a comprehensive understanding of the complex nature of pelvic pain, including its diverse etiologies, overlapping symptoms, and the importance of a biopsychosocial approach to care.^38^ This educational gap often leads to misdiagnosis, delayed diagnosis, and inappropriate or ineffective treatment.^10^, ^11^, ^39^ Women's pain is frequently dismissed or minimized, often by frustrated providers, resulting in patient frustration and a sense of being unheard, further compounding their suffering.^28^, ^40^ Inadequate education may also result in inequitable care, fragmented approaches, and, ultimately, poor patient outcomes.^11^ Fortunately, deficiencies in training can be addressed by improving training resources and investing in robust, standardized education for healthcare professionals across multiple disciplines, including primary care physicians, gynecologists, urologists, gastroenterologists, physical therapists, and mental health professionals.^11^, ^39^

## 7 NEED FOR PATIENT SUPPORT AND ADVOCACY

Women experiencing CPP often face a frustrating lack of accessible and reliable educational resources.^28^, ^34^ The complexity of CPP, coupled with the stigma surrounding pelvic health, makes it difficult for women to find clear information about their condition, understand treatment options, and navigate the healthcare system effectively.^33^, ^41^ This scarcity of patient‐centered resources leaves many women feeling isolated, confused, and disempowered.^3^, ^33^ Furthermore, while patient advocacy groups play a vital role in raising awareness and driving change, their reach and resources are often limited.^42^ Increased funding and support for patient education and advocacy resources are needed for empowering women with CPP, validating their experiences, and ensuring their voices are heard in research, policy, and healthcare delivery.^1^ Developing culturally sensitive and linguistically appropriate resources is also essential to address disparities in access to information and support.^1^, ^3^, ^15^

## 8 A CALL TO ACTION ON CHRONIC PELVIC PAIN

CPP inflicts significant suffering and economic burden upon millions of women worldwide, yet it remains a neglected area of health research and policy. This disparity between impact and resource allocation is unacceptable. This consensus statement calls for urgent action to prioritize CPP by promoting the following key actions:

- Establishing a consensus definition of chronic pelvic pain: the first step must be to obtain a consensus on the definition of CPP, agreed upon by multiple societies and advocacy groups. A clear definition paves the way for the development of a classification system, akin to PALM‐COEIN^43^ for abnormal uterine bleeding, which will enable clinicians to accurately diagnose CPP and enhance trainee and health professional education.^43^ An established definition will also assist researchers to precisely identify study populations, promote standardized data collection, and aid with the interpretation of results across studies, including systematic reviews and meta‐analyses. A clear definition empowers patients by giving them a name for their condition, validating their experience, and facilitating communication with healthcare professionals. Finally, a consensus definition provides policymakers with a focus for understanding the scope of the problem and allocating resources appropriately.
- Empower women and reduce stigma: women with CPP play a vital role in changing the landscape of care for this condition. Their lived experiences are invaluable in identifying gaps in care, developing more effective and compassionate treatment approaches, and driving improvements in access to care. The International Federation of Gynecology and Obstetrics (FIGO) and International Pelvic Pain Society (IPPS) call on affiliated societies to partner with women with CPP and patient advocacy groups to develop and implement strategies that not only remove stigma and create a supportive environment where women feel empowered to seek help, but also actively engage them in promoting education and advancing research on this condition. Their participation in studies, sharing of experiences, and input on research priorities are vital for enhancing our understanding of CPP mechanisms, developing innovative diagnostic tools, discovering more effective treatments, and developing policies that improve care. This type of collaboration with patients can be fostered through research, awareness campaigns, educational initiatives, and open dialogue.
- Expand access to quality transdisciplinary care: increasing access to transdisciplinary care for women with CPP requires a multifaceted approach. First, improving physician education is essential, ensuring that healthcare professionals across various specialties (e.g. gynecology, primary care, physical therapy, mental health) are well‐versed in the complexities of CPP and the benefits of collaborative care. Simultaneously, developing practical tools and resources, such as standardized assessment protocols, referral pathways, and patient education materials, can facilitate coordinated care delivery. Disseminating evidence‐based clinical guidelines will ensure consistent, high‐quality care across different healthcare settings. Integrating CPP care into primary care settings will enable early diagnosis, timely referral to specialized services, and improved care coordination. Importantly, these resources must be adaptable to countries with varied cultures and socioeconomic means. It is imperative that healthcare professionals and models of care shift away from treating CPP as an acute condition to a chronic care model that prioritizes long‐term management, physical and emotional rehabilitation, and mental well‐being. Policies should encourage the establishment of transdisciplinary pain clinics or networks, where specialists can work together, to provide comprehensive and integrated care. Finally, an evidence‐based biopsychosocial and trauma‐informed approach must be incorporated into all aspects of evaluation and management. This approach recognizes that women with CPP come from diverse backgrounds and varying socioeconomic statuses, and may have experienced traumatic events such as war, poverty, or abuse, all of which can significantly influence their pain experience and response to treatment. By acknowledging these factors and shifting the focus from “curing” an acute problem to managing a long‐term health condition, healthcare providers can promote well‐being and create a safe and supportive environment, foster trust, and facilitate more effective, patient‐centered care.
- Enhance education and awareness: CPP is often misunderstood, with many women suffering in silence. To address this, public awareness campaigns are needed to educate women, their families, and communities about CPP symptoms, diagnosis, and treatment options. These campaigns must challenge the harmful notion that pelvic pain is an acceptable or normal part of being a woman. By reducing stigma and encouraging help‐seeking behavior, we can empower women to take control of their health and seek timely care. Simultaneously, healthcare providers need comprehensive education and training to effectively diagnose and manage CPP. This includes promoting a transdisciplinary approach, utilizing the biopsychosocial model, and ensuring cultural sensitivity in all patient interactions. Providing women with culturally relevant educational resources will further empower them to actively participate in their care and navigate the healthcare system with confidence.
- Prioritizing research and funding: there are multifactorial causes for CPP and all of them are under‐studied. FIGO and IPPS emphasize the urgent need to recognize the underestimated impact of CPP. This underestimation leads to inadequate funding, limited recognition within healthcare systems, and ultimately, suboptimal care for millions of women. To address this, a rigorous assessment of CPP's full economic burden, including the often‐overlooked costs of unpaid work and lost productivity, is crucial. This comprehensive understanding will inform resource allocation and prioritize effective interventions. Furthermore, research efforts must explore the knowledge gaps, stigma, and barriers to care that perpetuate the cycle of neglect. Increased investment in transdisciplinary CPP research is essential. This research should prioritize innovative diagnostic tools, non‐surgical interventions that are more easily accessible, tailoring treatments to individual needs, and therapies that improve all aspects of physical, mental, and emotional health. Including CPP in global health agendas and research funding priorities is not only a matter of justice for women but is also essential for the well‐being and economic prosperity of societies worldwide.
- Developing dedicated CPP sections within healthcare societies: all relevant healthcare societies, including those representing gynecology, urology, gastroenterology, primary care, pain management, physical therapy, and behavioral and mental health, are strongly encouraged to develop dedicated sections, committees, or working groups specifically focused on CPP.
- FIGO and IPPS are committed to universal gynecologic health coverage and work as hard on creating preventive care strategies as they do on access to the best medical, surgical, and emergency gynecologic care.

In summary, FIGO and IPPS unequivocally call for universal access to quality healthcare for all women with CPP. Achieving the goals outlined in this consensus statement will require a concerted and collaborative effort from all stakeholders, including scientific societies, healthcare providers, researchers, policymakers, patient advocates, and women with CPP themselves. We believe that a multi‐pronged approach, encompassing individual empowerment, healthcare system reform, and global policy commitments, is essential for driving meaningful change. Through collaborative action and a shared dedication to improving women's health, we can reduce the global burden of CPP and enhance the well‐being of millions of women worldwide (Table 1).

**TABLE 1:** Call to action priorities.

Key actions	Outcomes
Establish a consensus definition of CPP	Allow for classification for researchers and healthcare providers Empower women by providing a diagnosis Provide policymakers with scope of the issue
Empower women and reduce stigma	Improves access care Encourages advocacy Drives the research agenda Opportunity to disseminate education
Expand access to quality transdisciplinary care	Move to a chronic care model Avoid unnecessary tests and interventions Use a patient‐centered approach
Enhance education and awareness	To the public To healthcare providers
Prioritize research and funding	Bridge knowledge gaps Develop innovative therapies with measured outcomes Use evidence to guide practice
Develop dedicated CPP sections within healthcare societies	Recognize the complexity of CPP Establish centers for provider training Encourage universal healthcare access

Abbreviation: CPP, chronic pelvic pain.

## AUTHOR CONTRIBUTIONS

All authors contributed to the conception, design, writing, and review of the paper. All authors agree to be accountable for the final publication.

## FUNDING INFORMATION

None.

## CONFLICT OF INTEREST STATEMENT

The authors have no conflicts of interest.

## Data Availability

Data sharing is not applicable to this article as no new data were created or analyzed in this study.

## References

[1] Lamvu G , Carrillo J , Ouyang C , Rapkin A . Chronic pelvic pain in women: a review. JAMA. 2021;325(23):2381‐2391. 34128995 10.1001/jama.2021.2631

[2] Chronic Pelvic Pain . ACOG practice bulletin, number 218. Obstet Gynecol. 2020;135(3):e98‐e109. 32080051 10.1097/AOG.0000000000003716

[3] Allaire C , Yong PJ , Bajzak K , et al. Guideline No. 445: management of chronic pelvic pain. J Obstet Gynaecol Can. 2024;46(1):102283. 38341225 10.1016/j.jogc.2023.102283

[4] Latthe P , Latthe M , Say L , Gülmezoglu M , Khan KS . WHO systematic review of prevalence of chronic pelvic pain: a neglected reproductive health morbidity. BMC Public Health. 2006;6:177. 16824213 10.1186/1471-2458-6-177 PMC1550236

[5] Margueritte F , Fritel X , Zins M , et al. The underestimated prevalence of neglected chronic pelvic pain in women, a nationwide cross‐sectional study in France. J Clin Med. 2021;10(11):2481. 34205077 10.3390/jcm10112481 PMC8199870

[6] Ahangari A . Prevalence of chronic pelvic pain among women: an updated review. Pain Physician. 2014;17(2):E141‐E147. 24658485

[7] Visceral and Other Syndromes of the Trunk Apart From Spinal and Radicular Pain . https://iaspfiles.s3.amazonaws.com/production/public/2021/Part_II‐F.pdf

[8] Raja SN , Carr DB , Cohen M , et al. The revised International Association for the Study of Pain definition of pain: concepts, challenges, and compromises. Pain. 2020;161(9):1976‐1982. 32694387 10.1097/j.pain.0000000000001939 PMC7680716

[9] Nicholas M , Vlaeyen JWS , Rief W , et al. The IASP classification of chronic pain for ICD‐11: chronic primary pain. Pain. 2019;160(1):28‐37. 30586068 10.1097/j.pain.0000000000001390

[10] Mardon AK , Leake HB , Szeto K , Moseley GL , Chalmers KJ . Recommendations for patient education in the management of persistent pelvic pain: a systematic review of clinical practice guidelines. Pain. 2024;165(6):1207‐1216. 38112691 10.1097/j.pain.0000000000003137

[11] Castellanos ME , Carrillo JF , Green I , Milspaw A , Lamvu G . Identifying gaps in pelvic pain education: a scoping review and structured analysis of obstetrics and gynecology training milestones. J Minim Invasive Gynecol. 2024;31(3):180‐192. 38081576 10.1016/j.jmig.2023.12.002

[12] Maixner W , Fillingim RB , Williams DA , Smith SB , Slade GD . Overlapping chronic pain conditions: implications for diagnosis and classification. J Pain. 2016;17(9):T93‐T107. 27586833 10.1016/j.jpain.2016.06.002 PMC6193199

[13] GBD 2019 Diseases and Injuries Collaborators . Global burden of 369 diseases and injuries in 204 countries and territories, 1990–2019: a systematic analysis for the global burden of disease study 2019. Lancet. 2020;396(10258):1204‐1222. 33069326 10.1016/S0140-6736(20)30925-9 PMC7567026

[14] Zondervan KT , Yudkin PL , Vessey MP , Dawes MG , Barlow DH , Kennedy SH . Prevalence and incidence of chronic pelvic pain in primary care: evidence from a national general practice database. Br J Obstet Gynaecol. 1999;106(11):1149‐1155. 10549959 10.1111/j.1471-0528.1999.tb08140.x

[15] As‐Sanie S , Black R , Giudice LC , et al. Assessing research gaps and unmet needs in endometriosis. Am J Obstet Gynecol. 2019;221(2):86‐94. 30790565 10.1016/j.ajog.2019.02.033

[16] Sewell M , Churilov L , Mooney S , Ma T , Maher P , Grover SR . Chronic pelvic pain—pain catastrophizing, pelvic pain and quality of life. Scand J Pain. 2018;18(3):441‐448. 29794266 10.1515/sjpain-2017-0181

[17] Demetriou L , Krassowski M , Abreu Mendes P , et al. Clinical profiling of specific diagnostic subgroups of women with chronic pelvic pain. Front Reprod Health. 2023;5:1140857. 37325239 10.3389/frph.2023.1140857 PMC10266100

[18] de Las Merces Villa RCY , Mazin SC , Nogueira AA , et al. Prevalence of chronic pelvic pain and primary dysmenorrhea in women of reproductive age in Ecuador. BMC Womens Health. 2022;22(1):363. 36056424 10.1186/s12905-022-01948-y PMC9438184

[19] Grol‐Prokopczyk H . Sociodemographic disparities in chronic pain, based on 12‐year longitudinal data. Pain. 2017;158(2):313‐322. 28092650 10.1097/j.pain.0000000000000762 PMC5242384

[20] Armour M , Lawson K , Wood A , Smith CA , Abbott J . The cost of illness and economic burden of endometriosis and chronic pelvic pain in Australia: a national online survey. PLoS One. 2019;14(10):e0223316. 31600241 10.1371/journal.pone.0223316 PMC6786587

[21] Tait RC , Chibnall JT . Racial/ethnic disparities in the assessment and treatment of pain: psychosocial perspectives. Am Psychol. 2014;69(2):131‐141. 24547799 10.1037/a0035204

[22] Goree JH , Jackson J . Do racial and ethnic disparities lead to the undertreatment of pain? Are there solutions? Curr Opin Anaesthesiol. 2022;35(3):273‐277. 35671012 10.1097/ACO.0000000000001139

[23] Nour NM . Global women's health: progress toward reducing sex‐based health disparities. Clin Chem. 2014;60(1):147‐150. 24046203 10.1373/clinchem.2013.213181

[24] Graeve C , Gao G , Stephenson V , Helland R , Jeffery AD . Impact of chronic pelvic pain on quality of life in diverse young adults. Arch Gynecol Obstet. 2024;310(6):3147‐3156. 39441406 10.1007/s00404-024-07783-w

[25] Zondervan KT , Yudkin PL , Vessey MP , et al. Chronic pelvic pain in the community‐‐symptoms, investigations, and diagnoses. Am J Obstet Gynecol. 2001;184(6):1149‐1155. doi:10.1067/mob.2001.112904 11349181

[26] Bartley EJ , Alappattu MJ , Manko K , Lewis H , Vasilopoulos T , Lamvu G . Presence of endometriosis and chronic overlapping pain conditions negatively impacts the pain experience in women with chronic pelvic‐abdominal pain: a cross‐sectional survey. Womens Health Lond. 2024;20:17455057241248017. 38682290 10.1177/17455057241248017 PMC11057341

[27] Barnes WA , Carter‐Brooks CM , Wu CZ , Acosta DA , Vargas MV . Racial and ethnic disparities in access to minimally invasive gynecologic surgery for benign pathology. Curr Opin Obstet Gynecol. 2021;33(4):279‐287. 34016820 10.1097/GCO.0000000000000719

[28] Hutton D , Mustafa A , Patil S , et al. The burden of chronic pelvic pain (CPP): costs and quality of life of women and men with CPP treated in outpatient referral centers. PLoS One. 2023;18(2):e0269828. 36757947 10.1371/journal.pone.0269828 PMC9910684

[29] Green I , Briggs M , Encalada Soto D , et al. Health care utilization by patients with chronic pelvic pain. Obstet Gynecol. 2024;144(1):4‐11. 38696811 10.1097/AOG.0000000000005595

[30] Huang G , Le AL , Goddard Y , et al. A systematic review of the cost of chronic pelvic pain in women. J Obstet Gynaecol Can. 2022;44(3):286‐293.e3. 34587539 10.1016/j.jogc.2021.08.011

[31] Hoskyns C , Rai SM . Recasting the global political economy: counting women's unpaid work. New Political Econ. 2007;12(3):297‐317.

[32] Zondervan KT , Griffith LG , Horne AW , Hummelshoj L , Stratton P , Missmer SA . Women's health: tackle the research funding deficit. Nature. 2023;619(7969):252. 10.1038/d41586-023-02231-2 37433932

[33] Chen I , Money D , Yong P , Williams C , Allaire C . An evaluation model for a multidisciplinary chronic pelvic pain clinic: application of the RE‐AIM framework. J Obstet Gynaecol Can. 2015;37(9):804‐809. 26605450 10.1016/S1701-2163(15)30151-1

[34] Blumenthal D , Abrams MK . Tailoring complex care management for high‐need, high‐cost patients. JAMA. 2016;316(16):1657‐1658. 27669168 10.1001/jama.2016.12388

[35] Davenport S , Smith D , Green DJ . Barriers to a timely diagnosis of endometriosis: a qualitative systematic review. Obstet Gynecol. 2023;142(3):571‐583. 37441792 10.1097/AOG.0000000000005255

[36] Agarwal SK , Chapron C , Giudice LC , et al. Clinical diagnosis of endometriosis: a call to action. Am J Obstet Gynecol. 2019;220(4):354.e1‐354.e12. 10.1016/j.ajog.2018.12.039 30625295

[37] Becker CM , Bokor A , Heikinheimo O , et al. ESHRE guideline: endometriosis. Hum Reprod Open. 2022;2022(2):hoac009. 35350465 10.1093/hropen/hoac009 PMC8951218

[38] Witzeman K . Pelvic pain: the pain problem with No home. Pract Pain Manag. 2022;22(2).

[39] Witzeman KA , Kopfman JE . Obstetrics‐gynecology resident attitudes and perceptions about chronic pelvic pain: a targeted needs assessment to aid curriculum development. J Grad Med Educ. 2014;6(1):39‐43. 24701308 10.4300/JGME-D-13-00053.1 PMC3963792

[40] Mathias SD , Kuppermann M , Liberman RF , Lipschutz RC , Steege JF . Chronic pelvic pain: prevalence, health‐related quality of life, and economic correlates. Obstet Gynecol. 1996;87(3):321‐327. 8598948 10.1016/0029-7844(95)00458-0

[41] Burke E , Di Renna T , Mustafa N , et al. Empowered management for pelvic pain: the experiences of women with persistent pelvic pain participating in an online self‐directed self‐management program while they wait for interprofessional care. Womens Health (Lond). 2024;20:17455057231224960. 38279872 10.1177/17455057231224960 PMC10822071

[42] Till SR , Wahl HN , As‐Sanie S . The role of nonpharmacologic therapies in management of chronic pelvic pain: what to do when surgery fails. Curr Opin Obstet Gynecol. 2017;29(4):231‐239. 28604402 10.1097/GCO.0000000000000376

[43] Munro MG , Critchley HOD , Broder MS , Fraser IS , FIGO Working Group on Menstrual Disorders . FIGO classification system (PALM‐COEIN) for causes of abnormal uterine bleeding in nongravid women of reproductive age. Int J Gynaecol Obstet. 2011;113(1):3‐13. 21345435 10.1016/j.ijgo.2010.11.011

